# Immobilization of Lipase A from *Candida antarctica* onto Chitosan-Coated Magnetic Nanoparticles

**DOI:** 10.3390/ijms20164018

**Published:** 2019-08-17

**Authors:** Rodolpho R. C. Monteiro, Paula J. M. Lima, Bruna B. Pinheiro, Tiago M. Freire, Lillian M. U. Dutra, Pierre B. A. Fechine, Luciana R. B. Gonçalves, Maria C. M. de Souza, José C. S. dos Santos, Roberto Fernandez-Lafuente

**Affiliations:** 1 Departamento de Engenharia Química, Universidade Federal do Ceará, Campus do Pici, Bloco 709, Fortaleza 60455760, CE, Brazil; 2 Departamento de Química Analítica e Físico-Química, Universidade Federal do Ceará, Campus do Pici, Bloco 940, Fortaleza CEP 60455760, CE, Brazil; 3 Instituto de Engenharias e Desenvolvimento Sustentável, Universidade da Integração Internacional da Lusofonia Afro-Brasileira, Campus das Auroras, Redenção 62790970, CE, Brazil; 4 Departamento de Biocatálisis, ICP-CSIC, Campus UAM-CSIC, 28049 Madrid, Spain

**Keywords:** lipase A from *Candida antarctica*, chitosan, magnetic nanoparticles, characterization

## Abstract

In this communication, lipase A from *Candida antarctica* (CALA) was immobilized by covalent bonding on magnetic nanoparticles coated with chitosan and activated with glutaraldehyde, labelled CALA-MNP, (immobilization parameters: 84.1% ± 1.0 for immobilization yield and 208.0 ± 3.0 U/g ± 1.1 for derivative activity). CALA-MNP biocatalyst was characterized by X-ray Powder Diffraction (XRPD), Fourier Transform Infrared (FTIR) spectroscopy, Thermogravimetry (TG) and Scanning Electron Microscope (SEM), proving the incorporation of magnetite and the immobilization of CALA in the chitosan matrix. Besides, the immobilized biocatalyst showed a half-life 8–11 times higher than that of the soluble enzyme at pH 5–9. CALA showed the highest activity at pH 7, while CALA-MNP presented the highest activity at pH 10. The immobilized enzyme was more active than the free enzyme at all studied pH values, except pH 7.

## 1. Introduction

Chemical and enzymatic catalysts are both effective for industrial applications [1,2]; nevertheless, chemical catalysis possesses several drawbacks, such as high energy consumption, undesirable by-product formation and equipment corrosion [3,4]. Enzymatic catalysis, on the other hand, is not as energy-demanding due to the mild operating conditions. It is also selective and specific, preventing undesired modifications of substrate and formation of toxic by-products, and it also presents very low environmental impact [5,6,7,8,9].

Lipases (triacylglycerol acyl hydrolases, E.C. 3.1.1.3–IUPAC) belong to a group of enzymes whose physiological function is to catalyze the hydrolysis of oils and fats [10]; apart from hydrolysis, under micro-aqueous environment, they are able to catalyze reactions such as esterification, transesterification, alcoholysis, acidolysis, epoxidations, etc., [11,12,13,14,15]. Basidiomycetous yeast *Candida antarctica* produces two different types of lipases, lipase A (CALA) and lipase B (CALB) [16]. CALB is the most extensively studied lipase in chemistry and fine chemistry [17], food technology [18] and bioenergy [19]; its crystalline structure was first reported in 1994 [20], whereas CALA had its crystalline structure more recently reported [21]. CALA has some useful properties to catalyze reactions, including high thermostability and the ability to tolerate a wide range of pHs [22,23].

The use of enzymes in their free or soluble forms evidences some problems related to low operational stability, difficult recovery and subsequent reuse of these biocatalysts [24]. However, such problems can be overcome when appropriate immobilization techniques are employed [25]; furthermore, once immobilized, enzymes exhibit greater resistance to pH, thermal and storage variations [26,27,28,29,30,31].

Depletion of oil reserves and an increase in crude oil prices coupled with the demand to protect the environment against pollution caused by mineral lubricating derived from petroleum and its uncontrolled disposal have driven to a growing interest in the use of biolubricants [32,33,34]. Biolubricants are an alternative to traditional lubricants, since they have valuable physicochemical properties (high lubricity, high viscosity index, high flash point and low vapor pressure, for example); besides having biodegradability and low toxicity towards humans and Nature [35,36]. Biolubricants may be produced from various vegetable oils, and it is also possible to produce them from synthetic esters and oils of fossil origin that meet biodegradability and toxicity criteria [37,38].

Literature reports a wide application of vegetable oils in bioenergetic industry; however, comparatively, there are few studies on the use of vegetable oils for lubrication purposes. Most vegetable oils used for these latter purposes are part of the human food chain (e.g., sunflower, soybean, corn, palm and coconut), which makes them unsuitable in the long run [39,40,41,42]. Thus, it is necessary to turn towards alternative sources of raw materials to produce biolubricants, preferably based on non-edible oils. An underutilized feedstock for biolubricant production is tilapia oil (*Oreochromis niloticus*) [43], which is one of the most widely cultivated fish in the world. In Brazil, tilapia accounts for approximately 45.4% of freshwater aquaculture production. There are large amounts of fish waste, especially viscera, which correspond to 7.5 to 15% of the weight of the fish [44,45]. Most of the time, residues from fish farming activity are dumped in landfills or directly into bodies of water, causing environmental problems. In this sense, the use of these residues to produce biolubricants will thus cover two environmental issues: production of non-toxic lubricants and use of a contaminating residue [46,47]. One point that makes interesting this material in the context of the paper, is that biolubricants use to give high viscosity and makes the recovery of immobilized biocatalysts difficult.

In this context, association of enzymes with magnetic nanoparticles (MNPs) may allow an easy separation of the enzymatic biocatalyst from the reaction medium by exposure to an external magnetic field, enabling a subsequent reuse of the biocatalyst [48]. The employed iron magnetic nanoparticles are solid dispersion particulates that exhibit paramagnetic properties at typical sizes of 10 to 20 nm [49]. The use of MNPs as solid supports for enzymatic immobilization has attracted considerable interest in the last decades; furthermore, low porosity and high mechanical and thermal stability are important properties of these nanostructured magnetic supports [50,51,52].

These naked iron nanostructures tend to aggregate and to become oxidized. This produces the loss of paramagnetism and dispersibility [53]. To overcome such problems, aiming at enzymatic immobilization, the surface of this type of solid support has been functionalized with organic polymers, silica or metalorganic structures [54]. Among these materials, we would like to remark chitosan, an abundant biopolymer with good biocompatibility and low toxicity. Chitosan is a copolymer obtained from the deacetylation of chitin, being a weak base, water insoluble in neutral or basic medium or in organic solvents, but it is soluble in dilute acidic media (pH < 6.5) [55,56]. Chitosan has been used in a wide variety of industrial fields such as biomedicine [57], papermaking [58], wastewater treatment [59], agriculture [60] or enzyme immobilization [61,62], among others.

For enzyme immobilization, chitosan has been used as a functional material since it offers a unique set of characteristics: biocompatibility, biodegradability to harmless products, nontoxicity, physiological inertness, antibacterial properties, heavy metal ions chelation, gel forming properties and hydrophilicity [61]. As coating for iron MNPs, chitosan controls size distribution, and thus enables the colloidal stability of these nanostructures [39,63,64,65] It also adds some new groups to the surface of the nano particles, introducing primary amino and hydroxyl groups.

Enzymes can be attached on the support by physical or chemical bonds [66]. Glutaraldehyde is generally used as the activating agent to covalently immobilize enzymes, especially on aminated supports [67], due to its high versatility [68,69]. When it is used, glutaraldehyde reacts with primary amino groups of the enzyme and the support; thus, a heterofunctional support is generated, having both physical and chemical interaction capacities [70,71].

The main objective of this communication is to evaluate the immobilization of lipase A from *Candida antarctica* on chitosan-coated magnetic nanoparticles activated with glutaraldehyde, here named CALA-MNP. Finally, we will check the feasibility of recovering the enzyme after producing biolubricants using tilapia fish viscera oil as source of the fatty acids.

## 2. Results and Discussion

### 2.1. Immobilization Parameters

The immobilization parameters were evaluated after 3 hours of immobilization, employing a protein load of 1 mg of protein per g of support for the hydrolysis of *p*-NPB (0.5 mM). The reference enzyme solutions (an enzyme prepared under identical conditions to the free enzyme but in absence of support) maintained full activity suring all immobilization process. That way, immobilization yield could be calculated by the decrease of activity in the supernatant. For the covalent immobilization (Fe_3_O_4_@CHI–GLU–CALA), the immobilization yield was around 84, the theoretical activity was just over 200 U/g, the actual derivative activity was under 70. This gave an expressed activity of more than 95%, as it can be seen in Table 1, which also presents the immobilization parameters for the biocatalyst prepared using no glutaraldehyde activated particles (Fe_3_O_4_@CHI-CALA). The enzyme immobilized on glutaraldehyde activated nanoparticles performed better than the non-activated nanoparticles, showing that glutaraldehyde plays a significant role on enzyme immobilization.

Osuna et al. immobilized lipase from *Aspergillus niger* on magnetic nanoparticles coated with chitosan and activated with glutaraldehyde, achieving an immobilization yield of 90.1 ± 1.1% and a mass activity of 309.5 ± 2.0 U/g [72]; Santos et al. obtained an immobilization yield of 94.70 ± 1.37% for the immobilization of CALB on chitosan support activated with glutaraldehyde [25]. Therefore, the immobilization parameters obtained in this study are in agreement with those reported in the literature, especially for the immobilization yield.

Glutaraldehyde, under the conditions used, is able to modify all the amino groups of chitosan, which can react with the amino residues of the lipase [72,73]. Therefore, lipase can be covalently immobilized on magnetic nanoparticles coated with chitosan and activated with glutaraldehyde base linkage between the aldehyde group of glutaraldehyde and the terminal amino group of lipase.

Pinheiro et al. immobilized lipase B from *Candida antarctica* on chitosan beads, achieving an immobilization yield of 55.6 ± 1% and 91.0 ± 1% when glutaraldehyde was used as activating agent [62]. Similarly, in this communication, the support presented a better performance for immobilization when activated with glutaraldehyde, even though the results clearly show that the support is able to immobilize CALA via ionic exchange. The modification of chitosan also reduces water retention capacity and makes it more resistant, improving its performance [74].

### 2.2. Effect of pH on the Thermal Stability of CALA Biocatalysts

At 85 °C, CALA and CALA-NPM were evaluated for thermal and pH inactivation. The stability of CALA-NPM was the highest at pH 9 (*t*_1/2_ = 222 min) followed by that at pH 5 (*t*_1/2_ = 92 min) and at pH 7 (*t*_1/2_ = 62 min). Free CALA also presented the highest stability at pH 9 (*t*_1/2_ = 27 min), followed by pH 5 (*t*_1/2_ = 10.1 min) and pH 7 (*t*_1/2_ = 5.7), but with significantly lower performance compared to the immobilized enzyme, as it can be seen in Table 2.

CALA is quite stable over a relatively broad pH range (6–9) [75], being highly stable in its immobilized form and it can be used at elevated temperature for thousands of hours without any significant loss in activity [76]. At pH 7, both CALA and CALA-MNP presented the lowest half-lives (5.7 and 62 min, respectively). This may be caused by the use of sodium phosphate at pH 7, as recently reported, it has negative effects on lipase stability [77].

In this case, stabilization due to immobilization ranged from 8.2 to 10.8. The lower the enzyme stability, the higher the stabilization due to immobilization.

### 2.3. Effect of pH on CALA Biocatalysts Activity

**Figure 1:**
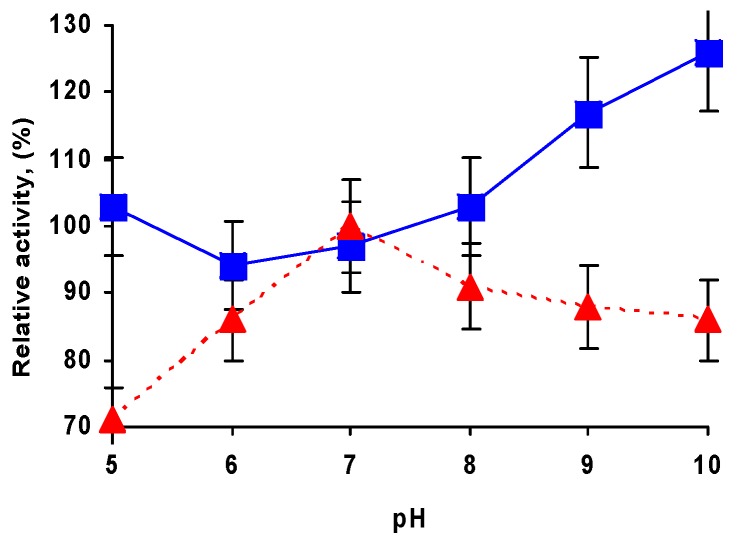
Effect of the pH value on *p*-NPB activity of CALA (red triangles) and CALA-MNP (blue squares). Further details are given in Section 3. 100% is considered the activity of the free enzyme at pH 7 (optimal conditions for the enzyme), and correspond to around 210 U/mg.

In order to evaluate the effect of pH on the biocatalysts performance, their activity was evaluated from pH 5 to pH 10, which are the limits of the substrate stability. As it can be seen in Figure 1, the soluble enzyme presented a quite flat profile, with a maximum at pH 7 and a lower activity at more acid or basic pH values. Other authors studied the effect of pH on the activity of CALA and observed the same profile obtained in this study, the highest activity was obtained at pH 7 [23,75,78].

For the immobilized enzyme, the effect of pH on the biocatalyst activity was fully different. In that case, a minimum activity at pH 6 was observed. Thus, although at pH 7 both immobilized and free enzymes have almost the same activity, under all the other pH values the immobilization of the enzyme increased significantly the specific activity of the enzyme (by a 50% at pH 9 or 10). Without discarding changes in the ionic interactions of the enzyme with the support that will depend on the pH value [79], this can be due to the increase in enzyme stability and/or conformational changes caused during immobilization.

### 2.4. Characterization of the Nanoparticles and Biocatalysts

**Figure 2:**
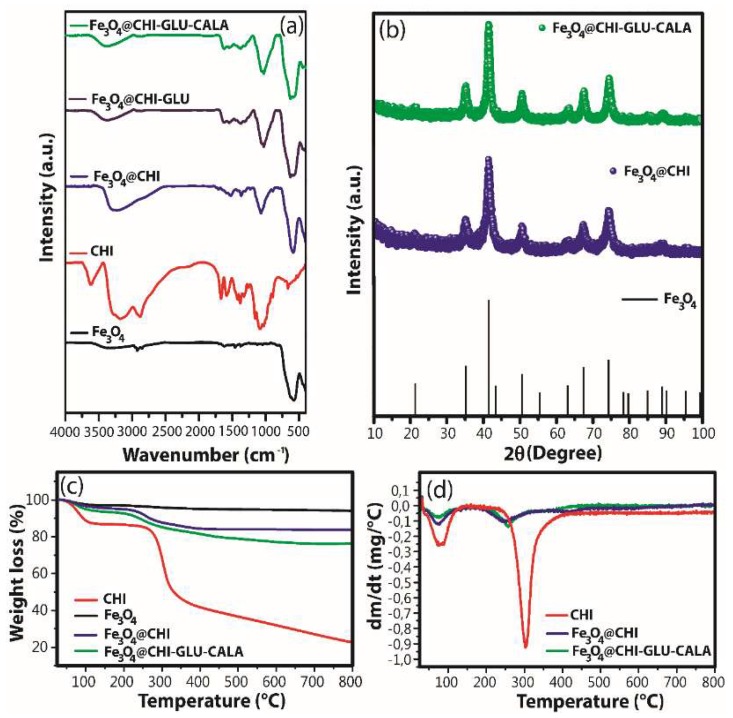
(**a**) FTIR, (**b**) XRPD, (**c**,**d**) TG and DTG of the synthesized samples.

In order to verify the incorporation of magnetite and the immobilization of CALA in the CALA-NPM, Fourier Transform Infrared (FTIR) spectroscopy absorption spectra were measured for the samples: Fe_3_O_4_, CHI, Fe_3_O_4_@CHI, Fe_3_O_4_@CHI–GLU and Fe_3_O_4_@CHI–GLU–CALA (Figure 2a). The crystalline structure present in the systems was determined by X-ray Powder Diffraction (XRPD). Figure 2b showed the profile diffraction peaks obtained for Fe_3_O_4_@CHI and Fe_3_O_4_@CHI–GLU–CALA. Thermogravimetry (TG) was used to evaluate the thermal stability and to quantify the relative organic amount in the nanocomposites (Figure 2c,d).

For the FTIR analysis, for Fe_3_O_4_, Fe_3_O_4_@CHI and Fe_3_O_4_@CHI–GLU–CALA samples, bands around 590 and 3340 cm^−1^ were respectively assigned to Fe–O deformation and O–H stretching vibration of water adsorbed on the magnetite surface [80]. A strong band around 590 cm^−1^ was not observed in CHI spectrum. Thus, this band can be used to confirm the presence of magnetite in the composites. In CHI, Fe_3_O_4_@CHI and Fe_3_O_4_@CHI–GLU spectra, C–O stretching vibration of glycosidic bonds was observed around 1075 cm^−1^ [81]. For CHI spectrum, the bands at 1319, 1379 and 1421 cm^−1^ can be referred to C–N stretching of amino groups and bending vibration of methylene and methyl groups, respectively [82,83]. For CHI and Fe_3_O_4_@CHI samples, the absorption around 1580 cm^−1^ is characteristic of –NH_2_ bending vibration [83]. This band was not observed in Fe_3_O_4_@CHI-GLU spectrum, suggesting that the crosslinking reaction between CHI and glutaraldehyde occurred. Additionally, the Fe_3_O_4_@CHI-GLU spectrum presents an absorption at 1622 cm^−1^ that can be assigned to C=O stretching vibration of amide groups and C=N of imine groups [64]. Interestingly, the spectra of Fe_3_O_4_@CHI-GLU and Fe_3_O_4_@CHI–GLU–CALA are very similar. However, the relative intensity of the band at 1622 cm^−1^ is greater in Fe_3_O_4_@CHI–GLU–CALA, suggesting an increase in the density of imine groups in its structure due to the crosslink reaction between CHI and CALA.

For XDR analysis, for Fe_3_O_4_@CHI and Fe_3_O_4_@CHI–GLU–CALA samples, peaks were identified at 21.3°, 35.3°, 41.3°, 50.7°, 63.° and 67.5° which can be assigned to (111), (220), (311), (400), (422) and (511) planes of Fe_3_O_4_ cubic spinel (ICSD, file-01-086-1340). Thus, it is important to note that the strategy used to synthesize nanocomposites was efficient, once the CHI coating can act as a protecting agent against magnetite oxidation and as site for lipase immobilization.

For TG analysis, in Figure 2c, the first thermal event, related to residual water loss, occurs between 30 and 125 °C for all samples [62]. For this event, the losses of 2.90%, 12.88%, 3.03% and 5.95% were observed for Fe_3_O_4_, CHI, Fe_3_O_4_@CHI and Fe_3_O_4_@CHI-GLU-CALA, respectively. In addition, DTG shows that the rate of degradation for this first thermal event increases following the order Fe_3_O_4_@CHI-GLU-CALA, Fe_3_O_4_@CHI and CHI. This can be assigned to the occupation of the hydrophilic sites of CHI, since the amine and hydroxyl groups must interact with the surface of magnetite and CALA, becoming less available to interact with water molecules [84]. The main polymeric degradation occurs in the temperature range of 200 to 414 °C for CHI and 180 to 493 °C for Fe_3_O_4_@CHI and Fe_3_O_4_@CHI–GLU–CALA [85]. The decrease of the polysaccharide thermal stability may be assigned to the breakdown of CHI–CHI intermolecular interaction, since the amine and hydroxyl groups of CHI, besides interacting with Fe_3_O_4_, participate of crosslinking reactions with the enzyme [86]. In DTG curves, the decrease in thermal stability is evident for CHI, since the maximum rate of the weight loss occurred at 302, 257 and 250 °C for CHI, Fe_3_O_4_@CHI–GLU–CALA and Fe_3_O_4_@CHI samples. Additionally, the weight loss for the second thermal event was 49.77, 11,17 and 14.10% for CHI, Fe_3_O_4_@CHI, and Fe_3_O_4_@CHI–GLU–CALA samples, respectively. For all the samples, the thermal degradation continues even in smaller proportion until 800 °C, corresponding to the maximum weight loss of 6.03, 77.33, 16.21 and 23.62% for Fe_3_O_4_, CHI, Fe_3_O_4_@CHI and Fe_3_O_4_@CHI–GLU–CALA samples, respectively. Since the sample Fe_3_O_4_@CHI–GLU–CALA presents a maximum weight loss greater than Fe_3_O_4_@CHI, it can be inferred that CALA was successfully incorporated on the CHI surface.

**Figure 3:**
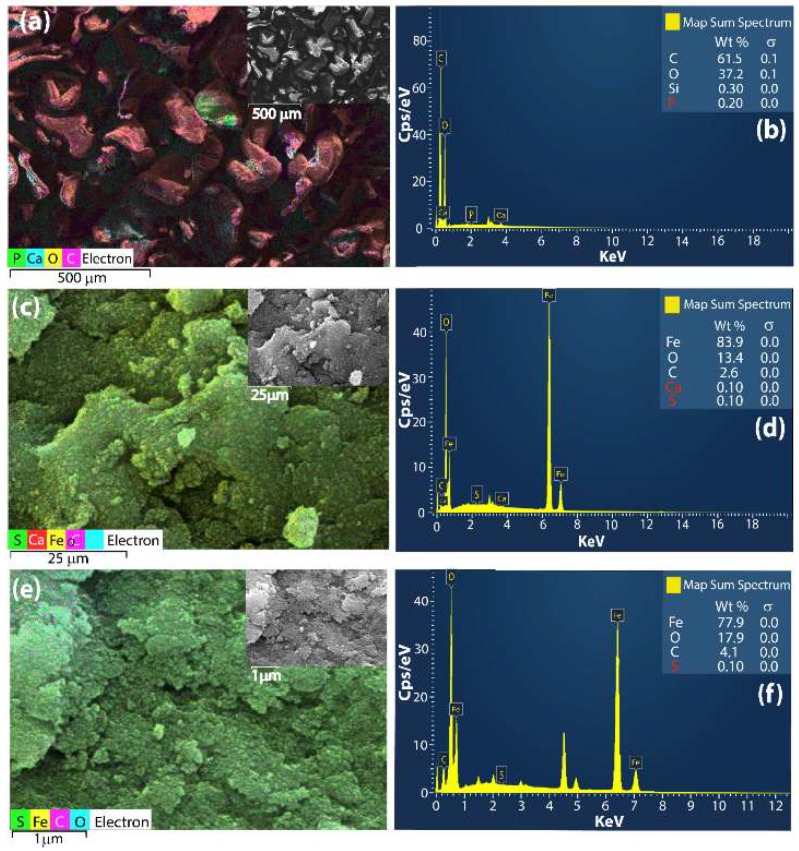
EDS maps, SEM images (inset) and EDS spectra of CHI (**a**,**b**), Fe_3_O_4_@CHI (**c**,**d**) and Fe_3_O_4_@CHI–GLU–CALA (**e**,**f**).

Scanning electron microscope (SEM) images of CHI, Fe_3_O_4_@CHI and Fe_3_O_4_@CHI–GLU–CALA are shown as inset Figure 3a,c,d. SEM images of CHI, Fe_3_O_4_@CHI and Fe_3_O_4_@CHI–GLU–CALA are shown as inset Figure 3a,c,d. These images illustrate the change that occurs on the CHI surface after the process of modification with Fe_3_O_4_. It was seen that CHI has a morphology similar to crushed shells (Figure 3a), with low roughness and porosity. After the incorporation of Fe_3_O_4_ in CHI matrix, the micrographs showed a huge change on the roughness, since the surface presents agglomerates that can be assigned to Fe_3_O_4_@CHI nanocomposite (Figure 3c).

**Figure 4:**
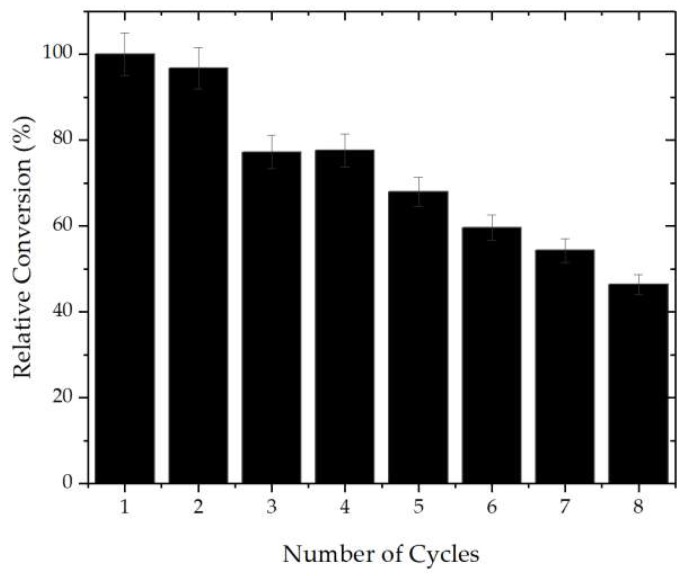
Operational Stability. Reaction medium: CALA-MNP = 208.0 ± 3.0 U/g, 2-ethyl-1-hexanol and free fatty acids from Tilapia oil (1:1) and 2% of content of biocatalyst. The reactions were performed for 24 hours at 30 °C at 200 rpm. Further details are given in Section 3.

No significant changes were observed on the nanocomposite surface after CALA immobilization (Figure 3e, inset). In order to evaluate the composition of the samples surface, EDS maps and EDS spectra were carried out (Figure 3a–f). From the EDS spectra, it was possible to obtain the weight percentage of the element on the surface of the nanocomposites. Figure 3d and Figure 4e showed that the surface of Fe_3_O_4_@CHI and Fe_3_O_4_@CHI–GLU–CALA samples is rich in iron with high homogeneity. For the CHI sample, no iron was observed in the EDS spectra (Figure 3b). Additionally, the percentage of carbon is higher in Fe_3_O_4_@CHI–GLU–CALA than Fe_3_O_4_@CHI. Thus, the EDS spectra suggesting that the CALA immobilization was successful, since the Iron/Carbon ratio decreased.

### 2.5. Operational Stability

Enzymes have higher prices than chemical catalysts; thus, to be competitive, immobilized enzymes should be able to be reused several times, maintaining thermal and operational stability [87]. Enzymes are very sensitive to changes in the environment; thus, several cycles of reactions may denature the protein [49], although its immobilization of enzymes promotes its rigidification, which keeps it active for longer periods of time, allowing consecutive reuse [88]. Using magnetic nanoparticles, and additional problem may be when recovering the biocatalysts in viscous solutions., like when a biolubricants is produced. Thus, we utilized the new biocatalysts in the production of biolubricants using 2-ethyl-1-hexanol and free fatty acids from Tilapia oil (1:1). These conditions permitted to get just around 25% conversion yield, but may be valid to check the possibility of the biocatalyst reuse. Prior to each cycle, the biocatalyst was separated from the reaction medium by magnetization and washed tree times with hexane to remove unreacted substrates. As it is shown in Figure 4, for the production of the biolubricant ester, the biocatalyst gave half of the initial conversion after 7 cycles of esterification. This was obtained even with so viscous reaction medium.

## 3. Materials and Methods

### 3.1. Materials

The commercial extract of lipase A from *Candida antartica* (CALA) (20.88 mg/mL) was obtained from Novozymes (Alcobendas, Spain). Iron (III) chloride hexahydrate, iron (II) sulfate heptahydrate, chitosan (50–190 kDa and 75–85% deacetylation degree), 25% (*v*/*v*) aqueous solution of glutaraldehyde, *p*-nitrophenyl butyrate (*p*-NPB), Triton X-100, 2-ethyl-1-hexanol (99.9%) were supplied by Sigma-Aldrich (St Louis, MI, USA); ammonium hydroxide (28–30%) was supplied by Dinâmica (São Paulo, Brazil). All other reagents and solvents used are of analytical grade. For the elaboration of the experimental design by the Taguchi method, Statistica^®^ 10 software (Statsoft, TULSA, OK, USA) was used and, for the creation of the graphics, OriginPro^®^ 2017 (OriginLab, Northampton, MA, USA) was used.

### 3.2. Methods

All experiments were performed at least in duplicate and the results are presented as the average of these values with the standard deviation.

#### 3.2.1. Synthesis of Iron Magnetic Nanoparticles (Fe_3_O_4_) Functionalized with Chitosan (CHI)

Iron magnetic nanoparticles (Fe_3_O_4_) functionalized with chitosan (CHI), (labelled Fe_3_O_4_@CHI in this study) were synthesized by the ultrasonic irradiation assisted by co-precipitation method. Briefly, 50 mg of CHI was suspended in 15 mL of an acetic acid (1%, *v*/*v*) solution. The resulting mixture was left under moderate stirring at 50 °C for 10 minutes. During this period, 10 mL of iron solution (0.33 M, FeCl_3_·6H_2_O/FeSO_4_·7H_2_O, following the molar ratio 2Fe^3+^:Fe^2+^) was added to the solution containing CHI. The final solution was homogenized by using an ultrasound probe with configuration of 20 s on, 10 s off and 50% amplitude. The solution was left for 4 min and then, still under ultrasound irradiation, 2 mL of NH_4_OH was slowly added and the system was kept under sonication for more 4 min. Once this was done, the precipitate was separated from the solution by magnetic decantation, washed several times with sodium phosphate buffer solution (25 mM and pH 7.0) to pH 7.0, and then dried and stored in a desiccator under vacuum [64].

#### 3.2.2. Activation of Fe_3_O_4_@CHI with Glutaraldehyde (GLU)

The amino terminal groups of Fe_3_O_4_@CHI were activated with glutaraldehyde (Fe_3_O_4_@CHI-GLU) to promote covalent bonding between the enzyme and the support. To do so, the protocol described by Xie et al., (2009) [89] was utilized, with some modifications. A volume of 25 μL of 25% (*v*/*v*) glutaraldehyde solution was placed in direct contact with 10 mg of previously dried Fe_3_O_4_@CHI. The mixture was kept under constant stirring for 2 h at 25 °C and was washed 3 times with 1 mL of 25 mM sodium phosphate at pH 7.0 to remove the excess of glutaraldehyde [89].

#### 3.2.3. Covalent Immobilization of CALA on Fe_3_O_4_@CHI-GLU

CALA (protein concentration 20.88 mg/mL in the commercial preparation) was immobilized on Fe_3_O_4_@CHI-GLU, the biocatalyst was named CALA-MNP. To do so, 100 mg of Fe_3_O_4_@CHI-GLU were suspended in 10 mL of 25 mM sodium phosphate buffer solution at pH 7.0 containing CALA (enzyme loading: 1 mg/g of support) in the presence of 0.01% Triton X-100. The system was kept under constant stirring for 3 h at 25 °C. An identical enzyme solution was prepared in absence of support, as a reference. In our experiments, the activity of this reference was maintained intact during the immobilization time. Finally, the immobilized lipase was separated from the solution by magnetic decantation and washed with 25 mM sodium phosphate buffer solution at pH 7.0 to neutrality. The amount of enzyme immobilized on the support was determined by measuring the initial and final activity of CALA in the supernatant of the immobilization suspension [90].

#### 3.2.4. Immobilization of CALA on Fe_3_O_4_@CHI

CALA was immobilized on Fe_3_O_4_@CHI (labelled Fe_3_O_4_@CHI-CALA in this study) by ionic exchange. A mass of 100 mg of Fe_3_O_4_@CHI-GLU was suspended in 10 mL of 25 mM sodium at pH 7.0 containing CALA (enzyme loading: 1 mg/g of support) in the presence of 0.01% Triton X-100. The immobilization process was similar to that described in the previous section.

#### 3.2.5. Determination of Enzymatic Activity and Protein Concentration

The activity of the soluble and immobilized enzyme was determined by the hydrolysis of *p*-nitrophenyl butyrate (*p*-NPB) as substrate; the released *p*-nitrophenol concentration was quantified spectrophotometrically at 348 nm. Activity measurements were performed in 25 mM sodium phosphate buffer solution at pH 7.0 and 25 °C, in the hydrolysis of 0.5 mM *p*-NPB ((isosbestic point, ᶓ = 5.236 mol^−1^.cm^−1^). [91]. To initiate the reaction, 50 μL of suspended lipase solution was added to 50 μL of *p*-NPB and 2.5 mL of the buffer solution. An international unit of activity (U) was defined as the amount of enzyme that hydrolyzes 1 μmol of *p*-NPB per minute under the conditions described above. The protein concentration was measured by the Bradford method [92] and bovine serum albumin was used as reference.

#### 3.2.6. Immobilization Parameters

The performance of the immobilized enzyme was evaluated by the immobilization parameters described by Silva et al. [56]. In short, the immobilization yield (IY) was defined as the ratio between the activity of enzymes retained on the support (initial activity – final activity) and initial activity (this is valid if the activity of the reference is maintained during the immobilization time). The theoretical activity (At_T_) was calculated using the immobilization yield (IY) and the enzyme load (it is the expected activity considering the activity of the free enzyme that has been immobilized). The recovered or expressed activity (At_R_) was defined as the ratio between the biocatalyst activity (_D_) and the theoretical activity (At_T_).

#### 3.2.7. Thermal and pH Inactivation

The biocatalyst was incubated in sodium acetate buffer (25 mM; pH 5.0), sodium phosphate buffer (25 mM; pH 7.0) or sodium carbonate buffer (25 mM; pH 9.0) at 85 °C. The activity was measured periodically using *p*-NPB and the residual activity was expressed as percentage of initial activity (hydrolytic activity before thermal incubation).

#### 3.2.8. Effect of pH on Biocatalyst Activity

The effect of pH on biocatalyst activity was evaluated; to do so, the biocatalysts were resuspended in 10 mL of sodium phosphate buffer (25 mM; pH 7.0). The activity was measured using *p*-NPB as described previously but using 25 mM buffers at pH varying from 5 to 10 (sodium acetate, sodium phosphate, and sodium carbonate). The activity was measured when the enzyme was added to the buffer and 15 min after. To ensure internal pH equilibration, the biocatalysts were incubated for 5 min in the measuring buffer before adding *p*-NPB [23].

#### 3.2.9. X-ray Powder Diffraction (XRPD)

XRPD analyses were carried out to confirm the crystalline structures in the samples. The analysis was carried out by X-ray powder diffractometer Xpert Pro MPD (Panalytical, Westborough, MA, USA) using Bragg–Brentano geometry in the range of 10°–100° with a rate of 1° min^−1^. CoKα radiation (*k* = 1.78896 Å) and the tube operated at 40 kV and 30 mA.

#### 3.2.10. Fourier Transform Infrared (FTIR) Spectroscopy

FTIR analyses were performed in a Perkin Elmer 2000 spectrophotometer used to record between 4000–400 cm^−1^. Previously, the samples were dried and pressed with KBr (~10 mg of sample to 100 mg of KBr) in disk format.

#### 3.2.11. Thermogravimetry (TG)

TGA were performed with 5 mg of nanoparticles under nitrogen atmosphere using a Thermogravimetric Analyzer Q50 V20. The loss of mass was monitored heating up samples from 25 to 800 °C in a rate of 10 °C min^−1^.

#### 3.2.12. Scanning Electron Microscope (SEM)

SEM images were performed on a SEM FEG Quanta 450 with EDS. Previously, the samples were deposited on a carbon tape and metalized with silver by the Metalizator Quorum QT150ES. 10 Pa of pressure was applied in the SEM chamber, with an incident electron beam of 20 kV.

#### 3.2.13. Extraction and Purification of Tilapia Oil

The extraction and purification of tilapia oil were performed according to methodology reported by Valle et al. (2013) [43]. In short, 1000 g of viscera of tilapia fish were placed under agitation at 900 rpm and heated at 80 °C for 40 min. Then, the mixture was filtered and decanted overnight. The upper phase (crude oil) was degummed with heated water (5% by weight) and, after centrifugation, the impurities were removed. It was necessary to perform neutralization of tilapia oil due to its high content of free fatty acids (7.2 mg KOH/g). To do so, 500 g of oil were placed under agitation at 250 rpm and heated to 60 °C and then an alkaline solution (4 g of potassium hydroxide and heated glycerin, 10% wt.) was added to the oil, the reaction was carried out for 15 min. After neutralization, the oil was centrifuged, washed with heated water (80 °C), dehumidified at 105 °C for 30 min, vacuum filtered with anhydrous sodium sulfate and stored at ambient temperature. The upper phase, rich in monounsaturated fatty acids, was used for the synthesis of biolubricant esters [43].

#### 3.2.14. Production of Free Fatty Acids (FFAs) from tilapia oil

The Free Fatty Acids (FFAs) of tilapia oil were obtained according to [93] with some modifications. In short, 100 g of purified oil and ethanolic KOH solution (3:1, alcohol/oil) were heated to 75 °C and reacted for 2 h under mechanical stirring at 400 rpm. The mass of KOH (40.6 g) used in the preparation of the ethanolic solution was determined from the saponification index value of the oil, with an increase of 10% to guarantee the complete reaction. At the end of the reaction, the mixture was transferred to a separatory funnel and washed with 6M HCl solution to pH 2.0. The upper oil phase, containing 200 mL of ethyl acetate, was washed with distilled water to neutral pH. The solvent was removed by rotary evaporator [93].

#### 3.2.15. Production of Biolubricant Ester

The production of the biolubricant ester was conducted in Eppendorf tubes (2 mL) in orbital shaker incubators at 200 rpm. The esterification was conducted using the FFAs obtained from the hydrolysis of the tilapia oil and 2-ethyl-1-hexanol as substrates, with molar ratio (acid:alcohol) 1:1. The reaction was started by adding 2% (mass of oil) CALA-MNP (and incubated at 3 for 24 h. After the specific reaction time for each assay, the acid index of the enzyme-free supernatant was determined for each sample. For this purpose, aliquots of 0.2 g were withdrawn from the reaction volume, diluted in 20 mL of ethyl alcohol and 3 drops phenolphthalein and then titrated with sodium hydroxide (40 mM) (Cavalcanti et al., 2018) [5], with some modifications. The acid index (AI) was established according to Equation (1) [5].

(1) $$AI\;\left(mgNaOH/g\right)=MM_{NaOH}\times {Щ}_{NaOH}\times f\times \left(\frac{V_{NaOH}}{m}\right)$$

where MM_NaOH_ (g/mol) is the molar mass of NaOH; Щ _NaOH_ (mol/L) is the molarity of the NaOH solution; *f* is the correction factor determined by NaOH standardization; V_NaOH_ (mL) is the volume of NaOH spent on the titration; and, m (g) is the mass of the sample to be analyzed. The conversion of FFAs to esters was calculated by Equation (2), considering the acidity at time zero (AI_0_) and time t (AI_t_) [5].

(2) $$Conversion\;FFA\left(\%\right)=\frac{AI_{0}-AI_{t}}{AI_{0}}\times 100$$

#### 3.2.16. Operational Stability

Operational stability was checked by consecutive reactions towards the production of the biolubricant ester using 2% for enzyme content, 1:1 for molar ratio (acid:alcohol), 30 °C for temperature and 24 h of reaction time stirred at 200 rpm. Prior to each cycle, the enzyme was separated from the reaction medium by magnetization and washed tree times with hexane to remove unreacted substrates [48].

## 4. Conclusions

Lipase A from *Candida antarctica* was immobilized on chitosan-coated magnetic nanoparticles of iron activated with glutaraldehyde. From immobilization parameters (84.1% ± 1.0 for immobilization yield and 208.0 ± 3.0 U/g ± 1.1 for derivative activity) to thermal and pH inactivation analysis (CALA-MPN half-life was more than 9 times bigger than that for CALA), the great performance of the immobilization procedure was established and confirmed by FTIR, XRPD, TG and SEM analysis.

## Figures and Tables

**Table 1:** Immobilization parameters of Fe_3_O_4_@CHI–GLU–CALA and Fe_3_O_4_@CHI-CALA: Immobilization yield (IY), theoretical activity (At_T_), actual biocatalyst activity (At_D_) and recovered activity (At_R_).

Biocatalyst	IY(%)	At_T_ (U/g)	At_D_ (U/g)	At_R_ (%)
Fe_3_O_4_@CHI–GLU–CALA	84.1 ± 1.0	212.2 ± 1.0	208.0 ± 3.0	98.0 ± 3.0
Fe_3_O_4_@CHI-CALA	44.3 ± 1.5	123.1 ± 1.5	120.8 ± 2.0	98.1 ± 2.0

**Table 2:** Half-life for CALA and CALA-MNP at 85 °C and pHs 5, 6 and 9.

Biocatalyst	Half-Life (*t*_1/2_, min)		
	pH 5	pH 7	pH 9
CALA	10.1	5.7	27
CALA-MNP	92	62	222
